# Effective treatment of *NR2F1*-related epilepsy with perampanel

**DOI:** 10.1186/s42494-023-00145-0

**Published:** 2024-01-24

**Authors:** Xiao Li, Kai Gao, Yutang Li, Yuehua Zhang, Han Zhang, Yuwu Jiang

**Affiliations:** 1 Department of Pediatrics, Peking University First Hospital, Beijing, 100034 China; 2 Beijing Key Laboratory of Molecular Diagnosis and Study On Pediatric Genetic Diseases, Beijing, 100034 China; 3 Key Laboratory for Neuroscience, Ministry of Education/National Health and Family Planning Commission, Peking University, Beijing, 100034 China; 4 Institute for Brain Disorders, Beijing, 100034 China; 5 Rehabilitation Department, Women and Children’s, Health Care Hospital of Yantai Zhifu, Yantai, 264000 China

**Keywords:** NR2F1, Epilepsy, Bosch-Boonstra-Schaaf optic atrophy syndrome, Perampanel, Infantile spasm

## Abstract

**Background:**

*NR2F1* mutations are associated with Bosch-Boonstra-Schaaf optic atrophy syndrome (BBSOAS). Although ~ 46.7% of BBSOAS patients present with epilepsy, which is always drug-resistant and associated with higher rates of behavioral and cognitive problems, the treatment and outcomes of *NR2F1*-related epilepsy have rarely been described. Here, we present new cases of BBSOAS-related epilepsy and summarize all previously reported cases to explore the effective treatment for this type of epilepsy.

**Methods:**

We identified six new Chinese cases of BBSOAS with epilepsy. Five different de novo heterozygous *NR2F1* mutations were identified in these cases, including two novel mutations c.365G > T, p.Cys122Phe and c.449G > T, p.Gly150Val. By combining the six cases and 14 previously reported cases, we analyzed the characteristics and treatment outcomes of *NR2F1*-related epilepsy.

**Results:**

Twelve of the 20 patients (60%) had infantile epileptic spasms, while the other patients had generalized tonic/tonic-clonic, focal, myoclonic, absence, or unclassified seizures. Several anti-seizure medications, steroids, and a ketogenic diet were administered in these cases. However, seizures were controlled in only 50% of previously reported cases, while all of the six new cases became seizure-free after perampanel as an add-on treatment. The average time from the addition of perampanel to seizure control was 7.33 ± 4.59 months (range, 1–12 months). The median time to seizure freedom was 14 months (1–32 months, > 19 months in 3 cases). The average dosage of perampanel needed for epilepsy control was 0.22 ± 0.17 mg/kg per day.

**Conclusions:**

In this paper, we comprehensively summarized the clinical characteristics, treatments and outcomes of *NR2F1*-related epilepsy for the first time. Perampanel exhibits dramatic efficacy for *NR2F1*-related epilepsy. This will help optimize the treatment of this type of epilepsy and provide clues for its pathogenic mechanisms. The two novel mutations expand the genotype spectrum of this disease.

**Supplementary Information:**

The online version contains supplementary material available at 10.1186/s42494-023-00145-0.

## Background

In 2014, *NR2F1* (nuclear receptor subfamily 2, group F, member 1) mutations were first associated with optic atrophy and intellectual disability by Bosch and Schaaf [1]. This disease was later named as Bosch-Boonstra-Schaaf optic atrophy syndrome (BBSOAS). BBSOAS symptoms include visual system defects, seizures, intellectual disability (ID)/developmental delay (DD), autism spectrum disorder (ASD), etc [1–4]. Due to the broad range of clinical abnormalities of BBSOAS, the presence of a *NR2F1* gene variant is the most crucial diagnostic indicator [3–8].

Epilepsy caused by *NR2F1* mutations was first reported in 2015, which expanded the clinical manifestations of BBSOAS [2]. To date, various types of epilepsy have been identified in patients with BBSOAS, including infantile epileptic spasms syndrome (IESS)/West syndrome [2–8]. Unfortunately, these *NR2F1*-related seizures are resistant to multiple anti-seizure medications (ASMs) and there were incomplete epileptic data at the time of reporting [5–8]. The presence of early-onset epileptic encephalopathy in infancy (primarily with IESS) is of particular importance as it is always associated with poor neurodevelopmental outcomes. Previously, ASMs for *NR2F1*-related epilepsy were selected according to the epilepsy syndrome and seizure type owing to the lack of specific and effective drugs.

In this study, we summarized the characteristics of 20 cases of *NR2F1*-related epilepsy with detailed epilepsy records (including our six new cases and 14 previously reported cases) to analyze the epileptic symptoms and treatment outcomes. In all of our six cases, perampanel effectively controlled the *NR2F1*-related epilepsy. The *NR2F1* pathogenic variants in our six cases are also provided, including two novel mutations, which expand the clinical phenotypes and genotypes of *NR2F1*-related epilepsy in BBSOAS.

## Methods

### Participants

**Fig. 1:**
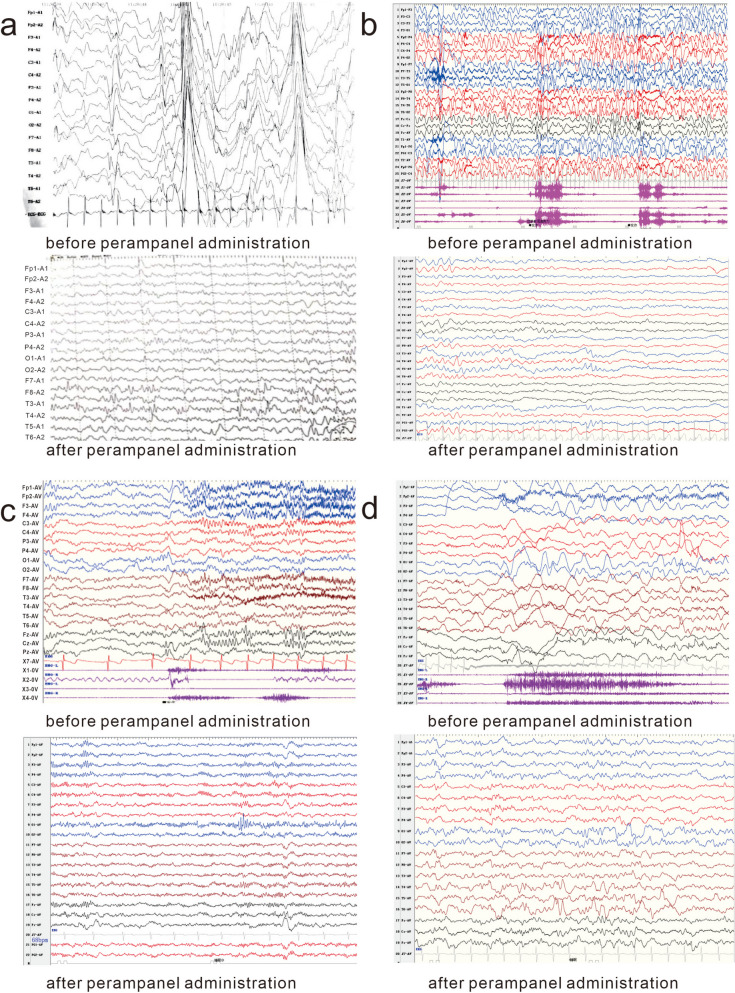
EEG recordings before and after administration of perampanel to Patients 1, 2, 3 and 5. **a**: In patient 1, before perampanel administration, bursts of high-amplitude spikes, multiple spikes and spike-slow waves, and multiple clinical attacks were detected (with a spasm of the whole body trunk) (age 5 months). At 15 months after perampandel administration, low-amplitude fast waves, spikes and spike-slow waves in were detected in the right middle and posterior temporal regions during sleep (age 2 years and 5 months). **b**: In patient 2, before perampanel administration, EEG recording detected hypsarrhythmia and bilateral discharges primarily in the posterior region (4 months); at 6 months after perampanel administration, no hypsarrhythmia, spike-slow waves, or sharp-slow waves were detected in the bilateral posterior regions (10 months). **c**: In patient 3, before perampanel administration, EEG recording detected spike-slow/sharp-slow waves, polyspike-slow/slow waves mainly in the posterior and the midline areas. Isolated and series of spasms as well as mild tonic attacks were detected (age 7 years and 6 months). At 4 months after perampanel administration, EEG recorded spike-slow/sharp-slow, slow, sharp, and spike waves mainly in the posterior and the midline areas (age 8 years). **d**: In patient 5, before perampanel administration, EEG recorded multifocal slow, spike and sharp waves, and one attack of focal origin (age 8 months). At 10 months after perampanel administration, 6-7 Hz waves with medium-to-high amplitudes and 15-20 Hz fast waves with a large number of continuous discharges were detected in the bilateral occipitotemporal regions (age 2 years and 7 months)

Six Chinese patients with epilepsy carrying *NR2F1* mutations were diagnosed with BBSOAS in our epilepsy clinic from 2018. Complete clinical data of epilepsy were available from all of the patients, including epilepsy characteristics, electroencephalogram (EEG) data, brain MRI scans, treatment procedure and prognosis. Another 14 cases of *NR2F1*-related epilepsy reported in literature were included in this study with detailed epileptic data, including epilepsy characteristics, EEG data, brain MRI scans, and sequencing information (Fig. 1).

### Ethics approval and consent to participate

This study was approved by the Clinical Research Ethics Committee of Peking University First Hospital. Written informed consent was obtained from the participants and their parents.

### Evaluation of ASM efficacy

Outcomes of ASM treatment were classified as seizure-free (seizures controlled completely, i.e., absence of hypsarrhythmia on EEG), ASM-responsive (seizures decreased by ≥ 50%), and no effect (seizures decreased by < 50%). In this retrospective study, the medication efficacy was assessed at 12 months after ASM treatment.

### Variant analysis

Five milliliters of peripheral venous blood were collected from patients and their parents. Genomic DNA was extracted for trio-based whole exome sequencing (WES). The pathogenicity of the variants was predicted by more than two bioinformatics programs, such as SIFT, PolyPhen-2, CADD, Protein Variation Effect Analyzer (PROVEAN), and MutationTaster, based on the American College of Medical Genetics and Genomics guidelines in 2015. Variants were evaluated against the general population using the ExAC, gnomAD, and dbSNP databases (Chigene company, Beijing, China). Sanger sequencing was performed for variant validation, and segregation analyses were performed following standard protocols.

### Statistical analysis

Continuous variables are reported as the mean ± standard deviation. Categorical variables are presented as frequencies and percentages. All analyses were conducted using the SPSS 19.0 software.

## Results

### Demographics

The mean age of the six cases was 46.5 ± 29.83 months (range, 20–104 months), and the male-to-female ratio was 5:1. The detailed clinical information is summarized in Table 1.

**Table 1:** Detail clinical data of the six BBSOAS patients in our clinic

ID	Age	Sex	*NR2F1* variant	Variant (Protein)	Brain MRI	DD/ID/ASD/ADHD/ Behavioral abnormalities	Visual System Defect(s) /Visual Deficit/VEP	Early-onset epilepsy/ seizures	Oromotor dysfunction
1	3 years and 10 months	Male	c.365G > T(NM_005654.4), de novo	p.Cys122Phe	Thin corpus callosum	Severe DD, ASD-like traits	P/SOD, alacrima, nystagmus and poor tracking, strabismus, poor tracking, VEP( +)	IES, GTS	NA
2	3 years and 5 months	Male	c.383G > T(NM_005654.4), de novo	P.Cys128Tyr	DM	DD, ASD-like traits	P/SOD, VEP( +), alacrima, nystagmus and poor tracking	IES	Yes
3	8 years and 8 months	Male	c.382 T > C(NM_005654.4), de novo	p.Cys128Arg	Normal	DD, ASD-like traits	Poor tracking, strabismus, VEP(NG)	IES, GTS, myoclonus	No
4	1 year and 8 months	Male	c.449G > T(NM_005654.4), de novo	p.Gly150Val	Normal	DD	Normal OD/ON, nystagmus, VEP( +)	IES	Yes
5	3 years and 5 month	Male	c.449G > T(NM_005654.4), de novo	p.Gly150Val	Thin corpus callosum	Developmental quotient 63, behavioral abnormalities	OA, strabismus, poor tracking, VEP(-)	IES, focal seizure	Yes
6	2 years 3 months	Female	c.328_330del(NM_005654.4), de novo	p.Phe110Del	Wider outer fronto-temporal space,thin intracranial segment of optic nerve	DD, behavioral abnormalities	Optic nerve pallor, rod cell dysfunction, strabismus, poor tracking, VEP( +)	IES, AS, myoclonus	Yes

*NA* not available, *ID* intellectual disability, *IES* infantile epileptic spasms, *AS* atonic seizure, *GTS* generalized tonic seizure, *DM* delayed myelination, *DD* developmental delay, *ASD* autism spectrum disorder, *P/SOD* pale/small optic disc, *VEP* visual evoked potentials, *OA* optic atrophy

### Epilepsy

To gain a better understanding of *NR2F1*-related epilepsy, we included only BBSOAS patients with seizures in our epilepsy clinic. The six patients had a mean epilepsy onset age of 3.08 ± 1.96 months (range, 0.5–6 months). The seizure types mainly included focal seizures, infantile epileptic spasms (IES), myoclonic seizures, atonic attacks, and generalized tonic seizures (GTS) (Table 2). IES, which is difficult to control, was observed in all six patients, with onset before 6 months of age.

**Table 2:** Seizure information of the six patients with BBSOAS in our clinic

Patient	Age at last follow-up	Age at SZ onset	Seizure type	Brain MRI	EEG	ASMs used	ASMs in use at last follow-up	Time from perampanel administration to seizure-free status	Epilepsy outcomes
1	3 years and 10 months	6 months	IES, GTS	Thin corpus callosum	Bursts of high-amplitude spikes, multiple spikes and spike-and-slow waves, and multiple clinical attacks (with a spasm of the whole body trunk) (at 5 months); Low-amplitude fast waves, spikes and spike-and-slow waves in the right middle and posterior temporal regions during sleep (at 2 years and 5 months);	Steroid, TPM, vitamin B6, perampanel	Perampanel (0.037 mg/kg/day), TPM (4.32 mg/kg/day)	1 months	Seizure-free for 32 months since 1 year and 2 months (seizures began to decrease 1 week after perampanel added)
2	3 years and 5 months	0.5 months	IES	DM	High-amplitude arrhythmia, bilateral discharges mainly in the posterior region (4 months); Slow spikes and spike-and-slow waves in the bilateral parietal, occipital and posterior temporal areas; a series of focal spasms were detected (6.5 months); No high-amplitude arrhythmia, spike-and-slow waves, sharp-slow waves in the bilateral posterior head (10 months)	ACTH, magnesium sulfate, vigabatrin, TPM, vitamin B6, KD, perampanel	Perampanel (0.06 mg/kg/day), TPM (11.9 mg/kg/day)	7 months	Seizure-free for 27 months since 1 year and 2 months (seizures began to decrease 2 weeks after perampanel added)
3	8 years 8 months	2 months	IES, GTS, myoclonus	Normal	Spike-slow/sharp-slow wave, multiple spike-and-slow/slow waves mainly in the posterior and the midline areas; isolated and series of spasms or mild tonic attacks (7 years and 6 months); Spike-and-slow/sharp-slow, slow waves, sharp, and spike wave rhythms mainly in the posterior and midline areas (8 years)	ACTH, vigabatrin, TPM, VPA, CZP, LEV, LTG, perampanel	Perampanel (0.238 mg/kg/day), LTG (withdrawing)	3 months	Seizure-free for 9 months since 7 years and 11 months (seizures began to decrease 6 week after Perampanel added)
4	1 year 8 months	4 months	IES	Normal	Hypsarrhythmia during awake time and sleep, with intermittent phenomenon; three episodes of isolated spasm (7.5 months)	LEV, ACTH, vigabatrin, TPM, vitamin B6, VPA, perampanel	Perampanel (0.214 mg/kg/day, increasing), VPA (withdrawing), TPM (2.68 mg/kg/day)	9 months	Seizure-free for 1 month since 1 year and 7 months (seizures began to decrease 8 weeks after Perampanel added)
5	3 years and 5 months	4 months	IES, focal seizure	Thin corpus callosum	Multifocal slow waves, spikes and sharp waves, and one attack of focal origin was detected (8 months); 6–7 Hz rhythm with medium-to-high wave amplitudes and a 15–20 Hz fast wave rhythm with a large number of continuous discharges in the bilateral occipitotemporal regions (2 years and 7 months);	VPA, TPM, LTG, perampanel	Perampanel (0.5 mg/kg/day), TPM (10 mg/kg/day)	12 months	Seizure-free for 19 months since 1 year and 10 months (seizures began to decrease 4 weeks after perampanel added)
6	2 years and 3 months	2 months	IES, AS, myoclonus	Wider outer frontotemporal space	Multifocal spike-and-slow/sharp-slow waves, spike waves, sharp waves in the bilateral posterior areas. Several spasms were detected, and atonic seizure followed myoclonic seizures (1 year and 6 months)	Perampanel	Perampanel (0.25 mg/kg/day)	12 months	Seizure-free for 9 months since 1 year and 8 months (seizures began to decrease 6 weeks after perampanel added)

*SZ* seizure, *IES* infantile epileptic spasms, *AS* atonic seizure, *GTS* generalized tonic seizure, *DM* delayed myelination, *ASMs* anti-seizure medications, *TPM* topiramate, *VPA* valproate, *CZP* clonazepam, *LEV* levetiracetam, *LTG* lamotrigine, *ACTH* adrenocorticotropic hormone, *KD* ketogenic diet

We reviewed all previously published BBSOAS cases to collect detailed clinical information (Additional file 1: Fig. S1; Table 3). One hundred and twelve BBSOAS patients with *NR2F1* mutations had been reported previously, of whom 45 BBSOAS patients were documented to have seizure attacks. However, only 14 individuals had available information on seizure type, seizure onset age, brain MRI scans, and EEG recording. The outcomes of epilepsy treatment were reported in eight of the 14 cases, and 4 of the 8 patients reported effective treatment with ASMs. Together with the six patients in our epilepsy clinic, a total of 20 BBSOAS patients were included for review (Table 3). The seizure types included IES, GTS, generalized tonic-clonic seizure (GTCS), focal seizures, myoclonus, absence, atonic, and some undetermined seizure patterns. Thirteen patients (65%) had IES, and the mean age of IES onset was 3.50 ± 1.54 months (range, 0.5–9 months).

**Table 3:** Seizure information of the 20 BBSOAS patients

Patient (age/sex)	*NR2F1* variant	Age at onset	Seizure type	Brain MRI	EEG	ASMs used	Effective ASMs	Epilepsy outcome	References
7 years/M	5q15 [88,945,075–134-105,929,496–555; 17 Mb]	6 years	GTCS	Bilateral PH, involving the temporal and occipital horns	Normal background activity with no epileptic-form discharges	NA	NA	Seizure-free	[9]
5 years/F	5q15 [87,086,298–35-95,538,640–699;8.4 Mb]	9 months	IES	Bilateral PH, involving the temporal and occipital horns	Poorly organized background activity and multifocal epileptic-form discharges	Resistant to anti-epileptic medication	NA	Not controlled	[9]
5 years/M	5q15 [88,659,488–547-94,986,541–600; 6.3 Mb]	8 months	Episodes of unresponsiveness, myoclonus	Bilateral PH, involving the temporal and occipital horns. Rotated hippocampi, more severely on the right, and irregular thickening and folding of the cortex in the posterior perisylvian regions, consistent with polymicrogyria	Bursts of multifocal and bilaterally synchronous epileptic-form activity	VPA	VPA	Seizure-free at 3 years	[9]
F	c.403C > T; p.Arg135Cys	4 months	IES, secondary generalized seizures	Normal	Hypsarrhythmia	NA	NA	NA	[2]
4 years/F	c.403C > A; p.Arg135Ser	4 months	IES	Thin corpus callosum	NA	Prednisone and OXC	NA	NA	[3]
6 years/M	c.328_330del; p.Phe110del	4 months	IES	Thin corpus callosum	Hypsarrhythmia	NA	NA	NA	[3]
21 years/M	c.1103G > A; p.Gly368Asp	18 years	Generalized seizure	Normal	Spike discharges in the paracentral and central areas	NA	NA	NA	[3]
43 years/M	C.2_4del; p.Met1?	13 years and 18 years	Two GTCS attacks	Normal	NA	VPA	VPA	Seizure-free at 18 years	[3]
7 years/M	c.328_330del; p.Phe110del	3 months	IES	Mild asymmetry of the lateral ventricle	Hypsarrhythmia and electroclinical spams	NA	NG	NA	[10]
23 years/F	c.403C > T; p.Arg135Cys	4 months	IES, GTS, GTCS, focal seizure	Normal		Pyridoxine, VPA, nitrazepam, steroid, and CZP	CZP,VPA	Seizure-free at around 20 years	[11]
14 years/M	c.257G > T; p.Cys86Phe	6 months	One-episode IES	Unremarkable and limited ophthalmologic evaluation disclosed bilateral mild optic nerve hypoplasia	Left-occipital-onset seizure with secondary generalization, hypsarrhythmia grade 3, generalized and multifocal spikes and sharp waves, and right temporal intermittent rhythmic delta activity	ACTH	ACTH	Seizure-free at 6 months	[12]
32 years/M	c.253 G > T; p.Glu85X	5 years	Episodes of behavioral arrest and non-responsiveness that lasted for 4 to 6 min	Unremarkable	Unremarkable	Phenobarbital, phenytoin, TPM, LEV, and LTG	TPM,LTG	Still on CZP	[5]
9 years/M	c.1080del; p.Asn362fs*33	2.5 years	Myoclonus, astatic seizure	Hypoplasia of the optic nerve and chiasma opticum as well as a hypoplastic corpus callosum	NA	Triple therapy	NA	Still having subcIinical epileptiform discharges at the last follow-up at age of 9 years and 10 months	[6]
20 years/F	c.2 T > C; p.Met1?	6 years	Episodic jerking movements	Markedly slender anterior visual pathways, almost complete absence of the septum pellucidum and possible truncation of the rostrum of the corpus callosum, highly suggestive of septooptic dysplasia	EEG at 12 years of age showed possible occipital seizures with repetitive high-amplitude spikes and slow waves bilaterally, with maximal activities in the posterior and occipital regions with a frequency of 2–4 per second	Clobazam	Clobazam (started on clobazam at age of 12)	NA	[13]
3 years and 10 months/M^a^	c.365G > T; p.Cys122Phe	6 months	IES, GTS	Thin corpus callosum	Bursts of high-amplitude spikes, multiple spikes and spike-and-slow waves, and multiple clinical attacks (with a spasm of the whole body trunk) (5 months); low-amplitude fast waves, spikes and spike-and-slow waves in the right middle and posterior temporal areas during sleep (2 years and 5 months);	ACTH, TPM, vitamin B6, and Perampanel	Perampanel	seizures-free	
3 years and 5 months/M^a^	c.383G > T; P.Cys128Tyr	0.5 month	IES	DM	High-amplitude arrhythmia, bilateral discharges mainly in the posterior area (4 months); slow, spikes and spike-and-slow waves in the bilateral parietal, occipital and posterior temporal areas; a series of focal spasms were detected (6.5 months); no high-amplitude arrhythmia, spikes-slow, sharp-slow wave in the bilateral posterior areas (10 months)	ACTH, Magnesium sulphate, Sabril, TPM, vitamin B6, KD, and Perampanel	Perampanel	seizures-free	
8 years and 8 months/M^a^	c.382 T > C; p.Cys128Arg	2 months	IES, GTS, myoclonus	Normal	Spike-and-slow/shar-slow waves, multiple spike-and-slow/slow waves mainly in the posterior and midline areas; isolated and series of spasms or mild tonic attacks (7 years and 6 months); spike-and-slow/sharp-slow, slow, sharp, and spike wave rhythm mainly in the posterior and midline area (8 years)	ACTH, Sabril, TPM, VPA, CZP, LEV, LTG, and Perampanel	Perampanel	seizures-free	
1 year and 8 month/M^a^	c.449G > T; p.Gly150Val	4 months	IES	Normal	Hypsarrhythmia during awake time and sleep, with intermittent phenomenon; 3 episodes of isolated spasm (7.5 months)	LEV, ACTH, Sabril, TPM, vitamin B6, VPA, and Perampanel	Perampanel	seizures-free	
3 years and 5 months/M^a^	c.449G > T; p.Gly150Val	4 months	IES, focal seizure	Thin corpus callosum	Multifocal slow waves, spikes and sharp waves, and one attack of focal origin (8 months); 6–7 Hz rhythm with medium-to-high wave amplitude and a 15–20 Hz fast wave rhythm with a large number of continuous discharges in the bilateral occipitotemporal regions (2 years and 7 months)	VPA, TPM, LTG, and Perampanel	Perampanel	seizures-free	
2 years and 3 months/F^a^	c.328_330; p.Phe110del	2 months	IES, AS, myoclonus	Wider outer frontotemporal space	Multifocal spike-and-slow/ sharp-slow waves, spikes, sharp waves marked in the bilateral posterior areas. Several spasms were detected, and atonic seizure followed myoclonic seizures (1 year and 6 months)	Perampanel	Perampanel	seizures-free	

*M* male, *F* female, *NA* not available, *PH* heterotopia, *SZ* seizure, *IES* infantile epileptic spasms, *AS* atonic seizure, *GTS* generalized tonic seizure, *GTCS* generalizedtonic-clonicseizures, *DM* delayed myelination, *ASMs* anti-seizure medications, *TPM* topiramate, *VPA* valproate, *CZP* clonazepam, *LEV* levetiracetam, *OXC* oxcarbazepine, *LTG* lamotrigine, *ACTH* adreno-cortico-tropic-hormone, *LEV* levetiracetam, *KD* ketogenic-diet

^a^Six new cases reported in this study

Since IES was the primary seizure type present in all of the six new cases, adrenocorticotropic hormone (ACTH), vigabatrin, vitamin B6, and topiramate (TPM) were first considered for treatment, followed by valproate (VPA), clonazepam (CZP), levetiracetam (LEV), and lamotrigine (LTG). A ketogenic diet was administered. The seizures were uncontrolled until the addition of perampanel (Fig. 1, Additional file 1: Fig. S4). After the addition of perampanel, all six (100%) patients became seizure-free. The average time from the addition of perampanel to seizure control was 7.33 ± 4.59 months (range, 1–12 months). The average time from perampanel addition to the appearance of effect was 4.5 ± 2.66 weeks. The median time to the seizure freedom status was 14 months (1–32 months, 3 cases > 19 months). The median follow-up time was 26 months (range: 11–32 months). The average perampanel dosage for epilepsy control was 0.22 ± 0.17 mg/kg per day. The DD/ID symptoms were improved in all six patients after seizure control.

According to the literature, ASMs used for *NR2F1*-epilepsy included VPA, oxcarbazepine, pyridoxine, nitrazepam, steroids, CZP, phenobarbital, phenytoin, TPM, LEV, LTG, and clobazam. Only four of eight patients (50%) with recorded ASM treatment became seizure-free. The effective ASMs in these four patients were VPA and ACTH, and the seizure types were GTCS, myoclonic seizures, absence seizures, and IES. Moreover, seven cases of IES have been reported previously, but only two had detailed treatment information. In one patient, the IES was controlled by ACTH application, whereas for the other patient, the seizures were uncontrolled and the detailed treatment data were not provided.

### Comorbidity

The most common clinical features across the six patients were epilepsy (6/6), DD/ID (6/6), vision impairment (6/6), hypotonia (6/6), and ASD-like traits (3/6).

Vision impairment was identified in all the patients by professional ophthalmologists. Three patients displayed optic atrophy (OA) or a pale/small optic disc. Four patients showed a prolonged latency and/or decreased amplitudes of visual-evoked potential (Table 1).

All of the six patients manifested moderate-to-severe DD/ID. Congenital developmental delay deteriorated after seizures, especially infantile spasms, and was improved after seizure control. Speech was affected profoundly as patients 3 and 5 could only make a sound resembling "mama" or "baba", and the other patients could just make babbling sound. The DD/ID symptoms of the 6 patients were all improved after seizure control. Patient 1 could sit and stand independently after seizure control. Patients 2 and 3 could not turn body over by themselves, but right after seizure control, they could sit independently and their cognitive ability was greatly improved. Patients 4 and 6 could walk with help, crawl independently and understand some simple commands after seizure control.

ASD is a major clinical comorbidity. Due to the profound DD/ID and young age, formal ASD evaluation was not applied, but ASD symptoms were observed in three of the six patients. Hypotonia was observed in all patients. Oromotor dysfunction, high pain tolerance, and common facial features such as prominent ears, epicanthal folds, a tall forehead, a thin upper lip, and downturned mouth corners were also observed in the six patients.

### Brain MRI

Brain MRI revealed a thin corpus callosum in two of our patients and non-specific slightly delayed myelination in one patient (Additional 1: Fig. S2). The other three cases had normal MRI finding.

### *NR2F1*-related genotyping

**Fig. 2:**
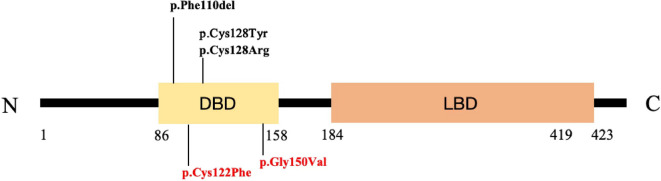
Distribution of the pathogenic variants in the Nr2f1 protein revealed in our cases. Two novel mutations are marked in red, including p.Cys122Phe in patient 2 and p.Gly150Val in patients 5 and 6. Abbreviations: DBD: DNA binding domain; LBD: ligand-binding domain

Five de novo heterozygous *NR2F1* mutations in the DNA-binding domain (DBD) were identified in our cases by trio-whole exome sequencing, including four missense mutations (at highly evolutionarily conserved residues across species) and one deletion mutation; two novel mutations were detected, including c.365G > T, p.Cys122Phe in one patient (Patient 1) and c.449G > T, p.Gly150Val in two patients (Patients 4 and 5) (Table 4, Fig. 2, Additional file 1: Fig. S3). Combined with the cases previously reported, there were 13 *NR2F1*-related patients with IESS (65%, 13/20). Twelve patients with IESS had mutations located in the DBD (nine missense mutations and three deletions), whereas the remaining patient had an 8.4-Mbp deletion in 5q14.3–15 [87,086298–95538640–699] (Table 1).

**Table 4:** *NR2F1* variants in the six patients with BBSOAS in our clinic

Patient	Age at last follow-up	Sex	*NR2F1* mutation	Heter/homo	Variant (Protein)	NR2F1 domain	dbSNP	Allele frequency ExAC/gnomAD (all)	In silico tools (PolyPhen-2, SIFT, MutationTaster, PROVEAN)	Reference
1	3 years and 10 months	Male	c.365G > T, de novo (chrM-14502 T > C, de novo)	heter	p.Cys122Phe	DBD	0/0	0/0	Damaging	-
2	3 years and 5 months	Male	c.383G > T, de novo	heter	P.Cys128Tyr	DBD	0/0	0/0	Damaging	[3]
3	8 years 8 months	Male	c.382 T > C, de novo	heter	p.Cys128Arg	DBD	0/0	0/0	Damaging	[3]
4	1 year 8 months	Male	c.449G > T, de novo	heter	p.Gly150Val	DBD	0/0	0/0	Damaging	-
5	3 years and 5 months	Male	c.449G > T, de novo	heter	p.Gly150Val	DBD	0/0	0/0	Damaging	-
6	2 years and 3 months	Female	c.328_330 del, de novo	heter	p.Phe110del	DBD	0/0	0/0	Damaging	[10]

*Heter/homo* Heterozygous/Homozygous, *del* deletion, *DBD* DNA binding domain, *SIFT* http://sift.jcvi.org/; Polyphen-2, http://genetics.bwh.harvard.edu/pph2/; ExAC Browser, http://exac.broadinstitute.org; gnomAD browser, http://gnomad.broadinstitute.org/

The three patients found with novel mutations all had IES, severe DD, visual problems, and swallowing disorders. Patient 6 also had a clear tendency towards self-harming and was extremely emaciated to need a gastric tube feeding.

## Discussion

*NR2F1* was first linked to OA with intellectual disability in 2014. This syndrome was named BBSOAS in a subsequent study. Although *NR2F1*-related epilepsy was first reported in 2015, most studies on *NR2F1* mutation-related BBSOAS have focused on visual impairment, ID/DD and psychobehavioral disorders. Less attention has been paid to related epilepsy.

Our results showed that the *NR2F1*-related epilepsies were mostly IESS (65%), which generally occurs within six months of life and is difficult to control. This type of epilepsy is always accompanied by visual impairment and ID/DD. Therefore, for patients with triad presentation including epilepsy, ID/DD, and vision defects, *NR2F1* sequencing should be conducted in a timely manner for early precision treatment.

We administered perampanel to patients 1, 2, 3, 4, and 5 who presented with seizures resistant to multiple treatments, and this drug showed dramatic efficacy in reducing the seizures. Based on this experience, the following patient 6 was treated with perampanel as an initial ASM when he was diagnosed with *NR2F1*-related epilepsy (DD, visual impairment, infantile spasm and WES presenting *NR2F1* de novo variant). This patient reached seizure-freedom with perampanel treatment alone within 12 months, and the seizure-free status remained 9 months later. In addition, we tried to withdraw ASMs except perampanel in patients 1, 2, 3, 4 and 5 after reaching the seizure-free status. The seizures relapsed in two patients during TPM withdrawal; therefore, we maintained TPM with perampanel in patients 1, 2, 4 and 5 while withdrawal of other ASMs continued. No recurrence or worsening of seizures occurred in these five cases. This indicates that perampanel combined with TPM may be an effective combinational regimen for *NR2F1*-related epilepsy**.**

We reviewed recent studies on the efficacy of ACTH, oral steroids or vigabatrin, which are the first-line treatments for IESS. In 2022, the National Infantile Spasms Consortium conducted a large-scale retrospective study on pediatric spasms in 23 medical centers in the United States. ACTH, oral steroids or vigabatrin was applied in 205, 99 or 91 children with IESS, respectively. Ultimately, the highest clinical remission rate was found at 30 days after treatment, which was, however, only 48% [14]. The latest randomized controlled trial on the standard treatment of IESS reported that, at 30 days after two-week treatment, 9/12 (75%) of the IESS children were controlled with ACTH, 1/9 (11%) with vigabatrin and 5/13 (38%) with combined therapy [15]. In 2018, 66 newly diagnosed patients with infantile spasms underwent sequential treatment and long-term follow-up. After 7 months of follow-up, a total of 48 patients (72.7%) were controlled [16]. However, there was no systematic research on the treatment of BBSOAS-related IESS epilepsy. In this retrospective study, we set 12 months as the time point for assessing medication efficacy, which is longer than the time frame in traditional studies of IESS. However, the pathology of BBSOAS-relate IESS is distinct from the general IESS, and vigabatrin and hormones can cause more severe long-term side effects than perampanel. Thus, considering the high seizure control rate in our patients (100%) compared with traditional treatments for corresponding epilepsy syndrome (e.g., ACTH and vigabatrin for IESS), we suggest that perampanel could be considered as the first-choice ASM for *NR2F1*-related epilepsy. Large prospective studies on the treatment of the BBSOAS-related IESS are needed in the future.

The pharmacological mechanism underlying the effectiveness of perampanel may be related to the pathogenicity of *NR2F1* mutations. NR2F1 has been verified to participate in many aspects of early nervous system development [17–24]. A review summarizing all the functional effects of reported *NR2F1* mutations proposed that *NR2F1* mutations identified in patients with BBSOAS mainly result in haploinsufficiency of the Nr2f1 protein [3, 4, 20]. Moreover, *NR2F1* variants in the DBD domain of the protein are thought to cause a greater loss of the transcriptional regulation function, possibly due to the dominant negative effect [20]. In 2020, Del Pino et al. identified that the loss of Nr2f1 protein in cortical progenitors resulted in increased intrinsic excitability in a mouse model [22]. Bertacchi et al. found that the brains of *NR2F1*-knockout mice display increased cortical plate thickness [8]. Conditional inactivation of Nr2f1 in mouse interneuron precursors results in decreased numbers of interneurons and aberrant migration [21]. Moreover, Teratani-Ota et al. reported that the majority of neuron-like cells generated from embryonic stem cells by Nr2f1 induction are GABA-positive and express other GABAergic neuronal markers [23]. These findings indicate that the normal expression of Nr2f1 is important for maintaining normal neurogenesis of GABAergic neurons and optimal excitement of cortical neurons. Thus, mutations of *NR2F1* may affect neurogenesis and the functions of GABAergic neurons, leading to an excessive increase in cortical excitability, and finally epilepsy. However, the first “patient-specific” NR2F1*-*R112K mutant mice generated by Zhang et al. [24] only recapitulated ASD-like traits while no epilepsy phenotype was observed. In summary, previous studies have indicated that the *NR2F1*-related epilepsy is primarily attributed to haploinsufficiency of the Nr2f1 protein caused by *NR2F1* variants, as Nr2f1 plays a role in maintaining the normal number and migration of interneurons, as well as the normal excitability of excitatory neurons in brain development. Consequently, decreased functionality of the Nr2f1 protein may lead to abnormal excitability of excitatory neurons as well as decreased number and aberrant migration of interneurons.

Perampanel selectively inhibits glutamate receptors [25, 26]. TPM is thought to exert antiepileptic effects through enhancement of GABAergic activity and inhibition of kainate/AMPA-type glutamate receptors [27]. The effectiveness of perampanel and TPM in our cases further supports the hypothesis that *NR2F1*-related epilepsy is due to the increased excitability and number of pyramidal neurons, as well as decreased number and aberrant migration of interneurons. To elucidate the mechanism of *NR2F1*-related epilepsy, experimental models with patient-specific *NR2F1* mutations, such as knock-in mouse models and brain organoids originating from patient-induced pluripotent stem cells, are needed.

A limitation of this study was that it was an observational and retrospective study without a randomized control group. We only recruited BBSOAS patients with *NR2F1*-related epilepsy; therefore, the number of patients recruited was relatively small. Moreover, all the six patients in our clinic suffered from IES. In future studies, patient data from multiple hospitals are needed to reduce the sampling error and focus on more types of *NR2F1*-related intractable epilepsy. Second, we did not conduct any experiments to verify our hypothesis that the effectiveness of perampanel on *NR2F1*-related epilepsy is due to the enhancement of pyramidal neurons and weakening of interneurons. Experimental models carrying patient-specific *NR2F1* mutations may be needed to fully explore the underlying mechanisms of *NR2F1*-related epilepsy.

## Conclusions

In summary, in this paper we summarize the clinical characteristics of *NR2F1*-related epilepsy, including treatments and outcomes. Infantile epileptic spasms was the most common seizure type of *NR2F1*-related epilepsy. Although *NR2F1*-related epilepsy was previously resistant to multiple anti-seizure medications and steroids, in our patients, perampanel exhibited dramatic effects on *NR2F1*-related epilepsy. This finding will help optimize the treatment of this type of epilepsy and provide insights into the pathogenesis of this epilepsy. We also reported two novel mutations, c.365G > T, p.Cys122Phe and c.449G > T, p.Gly150Val, which expand the genotype spectrum of this disease.

## Supplementary Information

**Additional file 1:** **Figure S1.** Flow chart of literature search. We searched the Pubmed, Embase and Cochrane Library databases using the following keyword combinations: (“NR2F1” and (“Bosch-Boonstra-Schaaf Optic Atrophy Syndrome” or ”BBSOAS”)), (“COUP-TFI” and (“Bosch-Boonstra-Schaaf Optic Atrophy Syndrome” or “BBSOAS”)), (“NR2F1” and ”epilepsy”), (“NR2F1” and “development”), (“Bosch-Boonstra-Schaaf Optic Atrophy Syndrome”) and (“NR2F1” and ”BRAIN”). Moreover, recently published reviews were screened to include additional records. **Figure S2.** Brian MRI and genetic mutation information of patient 2. Upper: brain MRI revealed delayed myelination of white matter in the bilateral insula (1 year and 3 months); Lower: Chromatograms of *NR2F1* mutation in patient 2. **Figure S3.** Species conservation analysis. **Figure S4.** Timelines of ASM adjustment for the 6 cases.

## Data Availability

All the data and materials are included in the paper and supporting files.

## Abbreviations

ASM: Anti-seizure medication
ASD: Autism spectrum disorder
ACTH: Adrenocorticotropic hormone
BBSOAS: Bosch-Boonstra-Schaaf optic atrophy syndrome
CZP: Clonazepam
DD: Developmental delay
DBD: DNA-binding domain
GTS: Generalized tonic seizures
GTCS: Generalized tonic-clonic seizure
ID: Intellectual disability
IESS: Infantile epileptic spasms syndrome
LEV: Levetiracetam
LTG: Lamotrigine
NR2F1: Nuclear receptor subfamily 2, group F, member 1
TPM: Topiramate
VPA: Valproate
WES: Whole-exome sequencing
