# Supplementary material for: Effective treatment of NR2F1-related epilepsy with perampanel
Source: Acta Epileptol. 2024 Jan 24;6:3. doi: 10.1186/s42494-023-00145-0 (PMC11960385; doi:10.1186/s42494-023-00145-0)
Supplement: Supplementary file 1 — Additional file 1: Figure S1. Flow chart of literature search. We searched the Pubmed, Embase and Cochrane Library databases using the following keyword combinations: (“NR2F1” and (“Bosch-Boonstra-Schaaf Optic Atrophy Syndrome” or ”BBSOAS”)), (“COUP-TFI” and (“Bosch-Boonstra-Schaaf Optic Atrophy Syndrome” or “BBSOAS”)), (“NR2F1” and ”epilepsy”), (“NR2F1” and “development”), (“Bosch-Boonstra-Schaaf Optic Atrophy Syndrome”) and (“NR2F1” and ”BRAIN”). Moreover, recently published reviews were screened to include additional records. Figure S2. Brian MRI and genetic mutation information of patient 2. Upper: brain MRI revealed delayed myelination of white matter in the bilateral insula (1 year and 3 months); Lower: Chromatograms of NR2F1 mutation in patient 2. Figure S3. Species conservation analysis. Figure S4. Timelines of ASM adjustment for the 6 cases. [file 42494_2023_145_MOESM1_ESM.docx]

Additional file 1


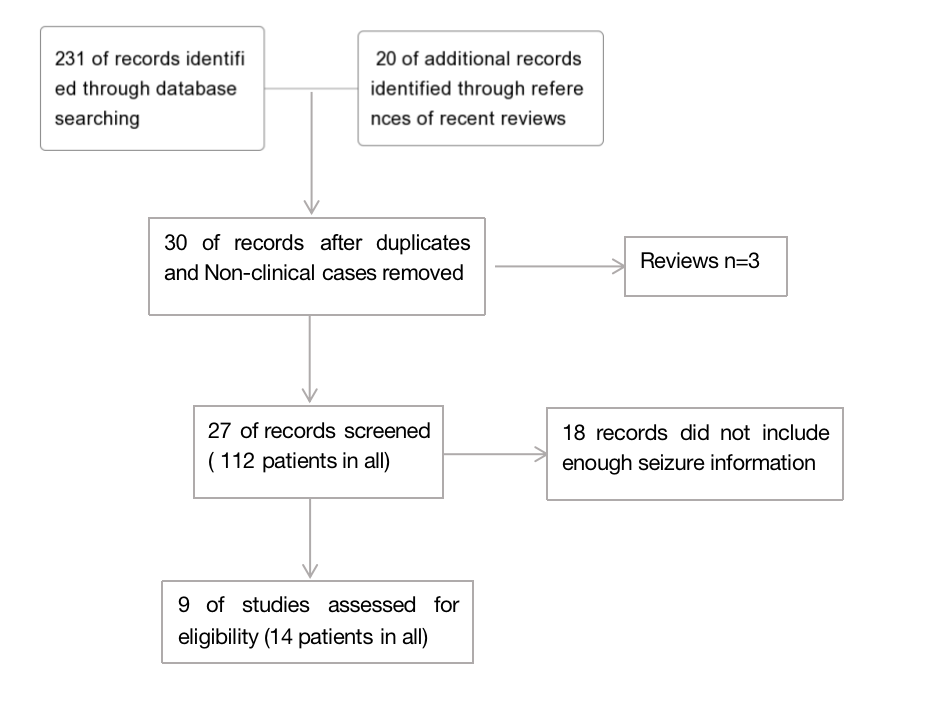


Figure S1. flow chart of literature search. We searched the Pubmed, Embase and Cochrane Library databases using the following keyword combinations: (“NR2F1” and (“Bosch-Boonstra-Schaaf Optic Atrophy Syndrome” or ”BBSOAS”)), (“COUP-TFI” and (“Bosch-Boonstra-Schaaf Optic Atrophy Syndrome” or “BBSOAS”)), (“NR2F1” and ”epilepsy”), (“NR2F1” and “development”), (“Bosch-Boonstra-Schaaf Optic Atrophy Syndrome”) and (“NR2F1” and ”BRAIN”). Moreover, recently published reviews were screened to include additional records.

**
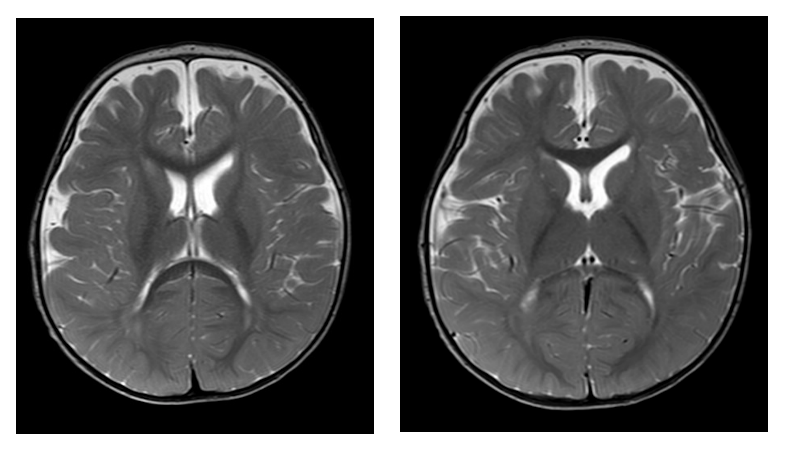
**
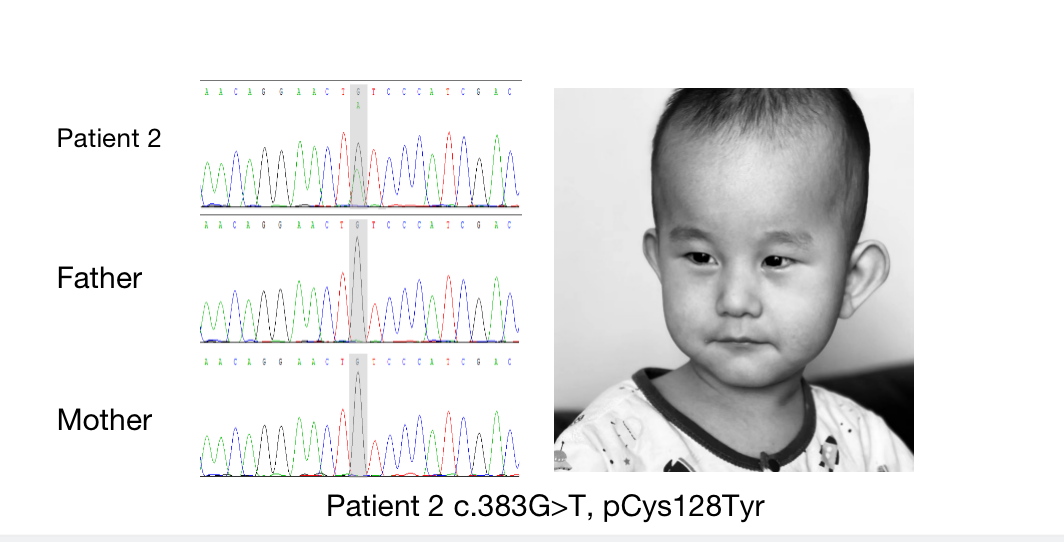


Figure S2. Brian MRI and genetic mutation information of patient 2. Upper: brain MRI revealed delayed myelination of white matter in the bilateral insula (1 year and 3 months); Lower: Chromatograms of *NR2F1* mutation in patient 2.


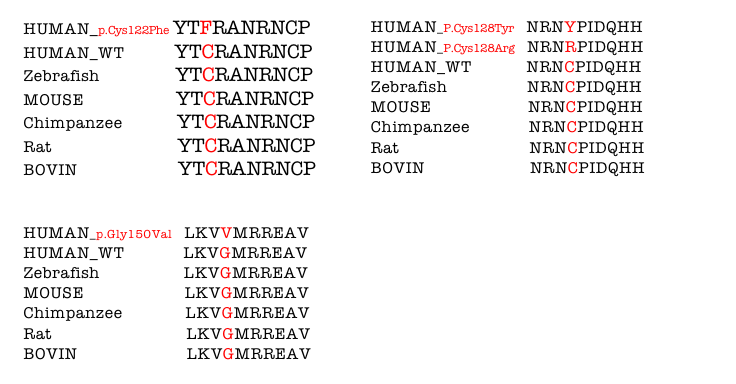


Figure S3. Species conservation analysis


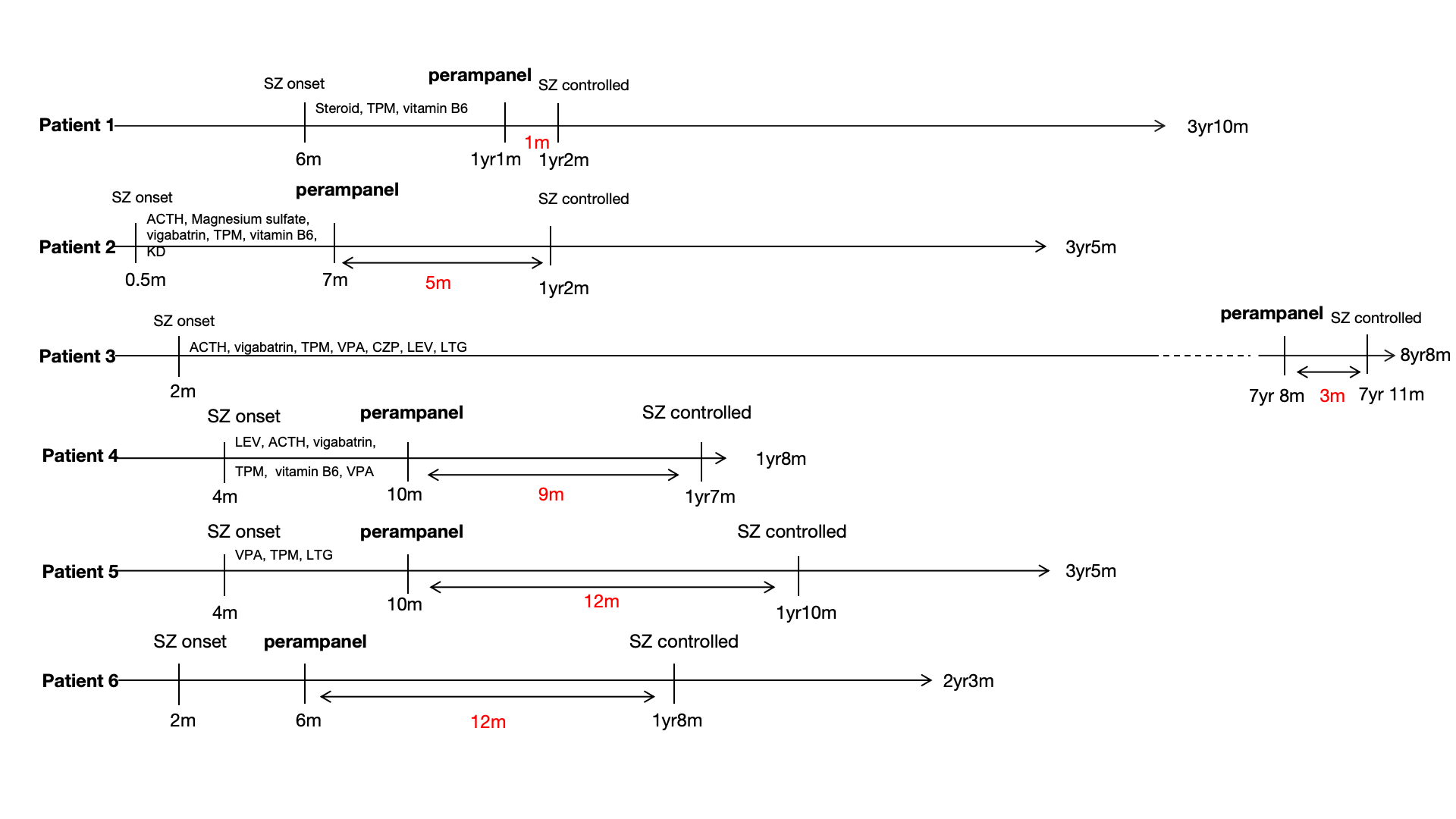


Figure S4. Timelines of ASM adjustment for the 6 cases
